# Evidence-based practice in neonatal health: knowledge among primary health care staff in northern Viet Nam

**DOI:** 10.1186/1478-4491-7-36

**Published:** 2009-04-24

**Authors:** Leif Eriksson, Nguyen Thu Nga, Mats Målqvist, Lars-Åke Persson, Uwe Ewald, Lars Wallin

**Affiliations:** 1 International Maternal and Child Health, Department of Women's and Children's Health, Uppsala University, Uppsala, Sweden; 2 Vietnam Sweden Uong Bi General Hospital, Quang Ninh, Viet Nam; 3 Neonatology, Department of Women's and Children's Health, Uppsala University, Uppsala, Sweden; 4 Department of Neurobiology, Care Sciences and Society, Division of Nursing, Karolinska Institutet, Stockholm, Sweden; 5 Clinical Research Utilization, Karolinska University Hospital, Stockholm, Sweden

## Abstract

**Background:**

An estimated four million deaths occur each year among children in the neonatal period. Current evidence-based interventions could prevent a large proportion of these deaths. However, health care workers involved in neonatal care need to have knowledge regarding such practices before being able to put them into action.

The aim of this survey was to assess the knowledge of primary health care practitioners regarding basic, evidence-based procedures in neonatal care in a Vietnamese province. A further aim was to investigate whether differences in level of knowledge were linked to certain characteristics of community health centres, such as access to national guidelines in reproductive health care, number of assisted deliveries and geographical location.

**Methods:**

This cross-sectional survey was completed within a baseline study preparing for an intervention study on knowledge translation (Implementing knowledge into practice for improved neonatal survival: a community-based trial in Quang Ninh province, Viet Nam, the NeoKIP project, ISRCTN44599712). Sixteen multiple-choice questions from five basic areas of evidence-based practice in neonatal care were distributed to 155 community health centres in 12 districts in a Vietnamese province, reaching 412 primary health care workers.

**Results:**

All health care workers approached for the survey responded. Overall, they achieved 60% of the maximum score of the questionnaire. Staff level of knowledge on evidence-based practice was linked to the geographical location of the CHC, but not to access to the national guidelines or the number of deliveries at the community level. Two separated geographical areas were identified with differences in staff level of knowledge and concurrent differences in neonatal survival, antenatal care and postnatal home visits.

**Conclusion:**

We have identified a complex pattern of associations between knowledge, geography, demographic factors and neonatal outcomes. Primary health care staff knowledge regarding neonatal health is scarce. This is a factor that is possible to influence and should be considered in future efforts for improving the neonatal health situation in Viet Nam.

## Background

The former executive director of UNICEF, James Grant, said: "The most urgent task before us is to get medical and health knowledge to those most in need of that knowledge. Of the approximately 50 million people who were dying each year in the late 1980s, fully two thirds could have been saved through the application of that knowledge" [1]. Many years after Grant's statement, the use of appropriate knowledge remains a global problem, particularly in the area of child health care. Every year almost 10 million children die in the world [2], of whom around four million die during the neonatal period [3]. This tragedy continues to unfold despite the existence of cheap, evidence-based interventions that could prevent a large proportion of these deaths [4].

Evidence-based practice (EBP) is a term increasingly used to describe the application of empirically acquired knowledge in practice [5,6]. In the neonatal period more than 70% of the current deaths could be prevented through evidence-based procedures (e.g. by exclusive breastfeeding and hypothermia management) [7]. However, health care workers involved in neonatal care need to have adequate knowledge about the different procedures before they can implement and use them. Educational programmes targeting health care staff in developing contexts have shown improvements in both staff knowledge and health care outcomes [8,9]. Thus, a primary issue is whether staff has the required knowledge or not. Understanding the level of knowledge is of interest for deciding what implementation strategy might be effective. Unfortunately, effective and sustainable implementation of knowledge into practice is not a trivial task, and only a few studies have evaluated strategies for knowledge translation in low-income countries [10-12].

Staff knowledge regarding evidence-based practice is key, but also a number of contextual factors are highly influential for a well-functioning health care system, such as adequate geographical coverage of health care, sufficiency of material resources (e.g. equipment and drugs) and a certain level of activity (e.g. number of assisted deliveries) at the health care units. Although the impact of contextual factors in relation to knowledge translation has been given a great deal of attention over the years [13,14], this has primarily been from the perspective of the local work context (e.g. leadership and workplace culture). Factors such as geographical location of health care units [15,16] and level of activity [17] have received less attention in relation to knowledge translation.

Viet Nam has achieved substantial improvements in child and infant survival, reporting a level of infant mortality corresponding to middle-income countries [18]. However, neonatal mortality has remained unchanged over the past three decades, currently constituting nearly three quarters of all infant deaths [19]. In 2003, the Ministry of Health in Viet Nam adopted a groundbreaking initiative to improve neonatal health care by launching practice guidelines for reproductive health care (here called the National Guidelines) [20]. These guidelines were disseminated to all public health care units providing antenatal, intrapartum and postnatal care, but were not accompanied by specific implementation activities.

In Quang Ninh province, our research group has set up the *Neonatal Knowledge Into Practice *project (NeoKIP, ISRCTN44599712). NeoKIP entails collaboration between Uppsala University in Sweden, the Ministry of Health in Viet Nam and the Viet Nam-Sweden hospital in Uong Bi, Viet Nam. The aim of NeoKIP is to evaluate facilitation; a knowledge translation intervention that we hypothesize will speed up identification of local health care-related problems at community level, increase primary health care staff knowledge and use of evidence-based knowledge and subsequently achieve improvement of neonatal outcomes.

In 2006, we performed a baseline study that identified an overall neonatal mortality rate (NMR) of 16 deaths per 1000 live births, with districts within the province ranging in NMR from 10 to 45 per 1000 [21]. The higher rates were noted in remote and mountainous districts, which are known to have a higher prevalence of poverty and people belonging to ethnic minority groups [22]. The existence of inequities in child survival is a well-known problem throughout the world and one on which more studies are needed to assess specific approaches to overcome these inequities [23]. Knowledge regarding evidence-based practice and use of this knowledge are central components for changing the severe situation. In the NeoKIP project, assessing knowledge will be helpful for planning and evaluating the coming intervention.

The aim of this survey was to assess the knowledge of primary health care practitioners regarding basic, evidence-based procedures in the neonatal care field in a Vietnamese province. Further aims were to assess the availability of material resources at the community health centres (CHCs) and to investigate whether differences in knowledge level were linked to (CHCs): (1) access to National Guidelines, (2) number of assisted deliveries and (3) geographical location.

## Methods

### Setting

The Quang Ninh province in Viet Nam is situated 120 km east of the Vietnamese capital, Hanoi, along the coast in the north-eastern corner of Viet Nam bordering China. Quang Ninh has approximately one million inhabitants living in an area of 5900 km^2^. The province is a mixture of urban, rural and mountainous settings. Coal mining is the most important industry, together with a rapidly growing tourism sector. More than 80% of the population belongs to the Kinh ethnic group, while most of the remaining individuals belong to five ethnic minority groups.

In Quang Ninh there are 14 districts that include 184 communities. Eighteen hospitals serve the province, of which one provincial hospital and one regional hospital are at tertiary level. In each community there is at least one CHC responsible for primary health care. The CHCs provide antenatal care (ANC), assistance in uncomplicated deliveries and newborn care. The CHCs are staffed by physicians, midwives, assistant doctors and nurses.

### Study population and data collection

Information on health care resources (equipment and drugs), number of ANC visits among pregnant women, postnatal home visits by a CHC staff, number of deliveries and neonatal deaths were collected from all 14 districts. More details on the data collection on live births and neonatal deaths are published elsewhere [21,24]. Because of logistics, the knowledge survey was not conducted in two of the districts. Thus, 12 districts with 155 CHCs participated in the knowledge survey. In these districts, 657 health care workers were employed. Doctors, assistant doctors, midwives and nurses involved in deliveries and newborn care at the CHCs were targeted for the knowledge survey. The health workers on duty at the CHCs at the time of data collection in the NeoKIP's baseline study (n = 412) were asked to participate.

A questionnaire for assessing staff knowledge was developed by the research team. It consisted of 16 multiple-choice questions (Additional file 1) covering basic aspects of EBP in neonatal care. The following five areas were included in the knowledge survey: breastfeeding, immediate postnatal care, infection management, low birth weight management and postnatal home visits. The choice of topics was based on EBP as described in the National Guidelines [20] and in World Health Organization (WHO) recommendations on newborn health care [25]. The selection of questions under each topic was based on their relevance for neonatal survival but also on specific issues that we found had shortcomings in the study area when discussing with practitioners during the baseline study. The questionnaire was pilot-tested by nurses in Sweden and CHC staff in Viet Nam and revised accordingly.

Fifteen full-time project employees collected data from April to June 2006 for the NeoKIP baseline. At each CHC, a data collector handed out the questionnaire to survey participants, who responded individually without access to any information sources. Whether a CHC or a hospital had access to certain equipment, drugs and the National Guidelines was determined through a visual audit by a data collector using a checklist with 19 items. At CHCs and hospitals, data collectors met with health care staff (obstetric and paediatric department at hospitals) and checked registers for information about the facility and its health care statistics.

A Geographic Information System (GIS) was set up to map the location of the health care facilities. Geographical coordinates were collected using a GPS (Garmin GPS 60). Data were managed in Mapsource (version 6.0; Garmin International Inc., Olathe, Kansas, United States of America) and ArcGIS (version 9; ESRI, Redlands, California, United States of America).

### Data analysis

A maximum of 48 points could be obtained in the knowledge survey. Each of the 16 questions could generate three points; for a maximum score, the respondent had to fill in the correct alternative(s) required for each question. A scoring system was developed for calculation of points that included reductions for incorrectly marked alternatives; a question could not generate less than zero points, however. The questionnaire responses were entered and analysed in SPSS (version 14.0; SPSS Inc, Chicago, Illinois, United States of America). For statistical analysis, independent sample t-test, one-way ANOVA and χ^2^-tests were used. The results of each question are presented as percentages of the total number of potential points. The survey results were compared with the number of deliveries for 2005 at each CHC. For this purpose, the health centres were sorted into three arbitrary groups: 0, 1–24 and ≥ 25 deliveries. For determining distances between districts and the two hospitals at tertiary level, ArcGIS 9 was used; the existing road network was not considered.

### Ethical considerations

The Ministry of Health in Viet Nam, the Provincial Health Bureau in Quang Ninh and the Research Ethics Committee at Uppsala University, Sweden, approved the study. Participation in the survey was voluntary. The respondents were informed about the purpose of the survey and gave their consent to participate. Data have been handled with confidentiality.

## Results

**Figure 1:**
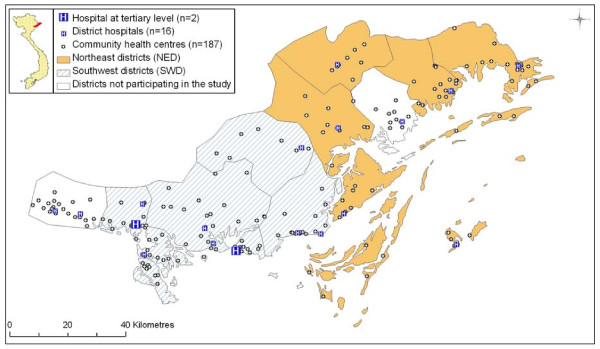
**Map over survey area**. Map over Quang Ninh province in northern Viet Nam indicating the location of hospitals and community health centres. Knowledge survey results indicated two areas of clustered districts: the northeast districts and the southwest districts.

Data collection was performed in all (n = 205) health care units (Fig. 1) providing neonatal care in Quang Ninh province. At tertiary level there were two hospitals and at district level 16 hospitals. The community level had 779 health care staff working in the 187 CHCs, including at least one midwife or one assistant doctor responsible for neonatal care at each CHC. The findings of the visual audit of 19 items for neonatal care revealed that most hospitals were well-equipped, whereas the CHCs generally were lacking in equipment and drugs for safe delivery, temperature control and neonatal resuscitation (Table 1).

**Table 1:** Availability of equipment and drugs at all hospitals and all community health centres in Quang Ninh province, Viet Nam

**Items**	**18 Hospitals % (n)**	**187 CHCs % (n)**
Guidelines		

The National Guidelines^a^	*67 (12)*	*70 (131)*


Hygiene and infections		

Soap	*100 (18)*	*94 (175)*

Clean gloves	*100 (18)*	*97 (181)*

Clean water	*100 (18)*	*81 (151)*^1^

Alcohol for disinfection	*94 (17)*	*95 (178)*

Iodine for disinfection	*100 (18)*	*92 (172)*

Antibiotics	*100 (18)*	*99 (185)*


Safe delivery		

Foetus stethoscope	*94 (17)*	*99 (185)*

Forceps	*44 (8)*	*2 (3)*^1^

Vacuum extraction equipment	*28 (5)*	*1 (2)*^1^

Vitamin K_1_	*67 (12)*	*11 (21)*^1^


Temperature control		

Radiant heater	*89 (16)*	*11 (21)*^1^

Towels for newborn	*78 (14)*	*38 (71)*^1^

Thermometer	*100 (18)*	*99 (185)*


Resuscitation		

Face mask and ambo for newborns	*89 (16)*	*10 (18)*^1^

Face mask and ambo for adults	*50 (9)*	*22 (41)*^1^

Manual suction	*17 (3)*	*16 (30)*

Suction machine	*94 (17)*	*76 (143)*

Tube for suction machine	*94 (17)*	*49 (92)*^1^

^a^*Guidelines of reproductive health *(2003) by the Ministry of Health in Viet Nam

^1^Difference (p < 0.05) in availability of an item compared with hospitals as derived from

the χ^2 ^test.

### Knowledge survey

**Figure 2:**
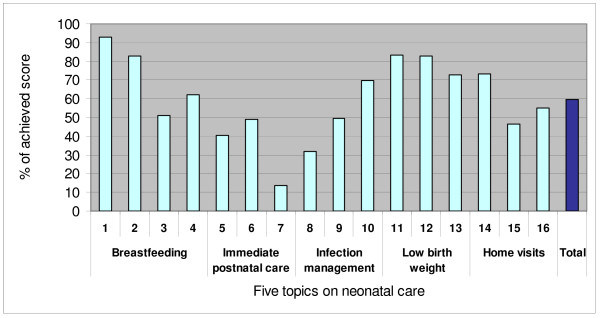
**Knowledge survey results**. Results of the knowledge survey among primary health care staff on five topics of neonatal health care (n = 412).

The questionnaire was completed by all (n = 412) primary health care workers on duty at the time of the knowledge survey, which was 63% (412/657) of the total number of staff in the 12 participating districts. Among the respondents, 8% (33/412) were doctors, 37% (151/412) assistant doctors, 24% (98/412) midwives and 31% (130/412) nurses. The mean age of the respondents was 37 years; 77% (316/412) were female and 80% (331/412) belonged to the Kinh ethnic group. In total, survey participants achieved 60% of the potential points (11 817 points out of 19 776) (Fig. 2), resulting in a mean score of 28.7 (SD ± 6.1) (11 817 points/412 participants). Individual results ranged from 3 to 44, and mean scores at the district level varied from 26.7 to 31.5. Midwives (30.4), medical doctors (29.2), nurses (28.7) and assistant doctors (27.4) differed in mean scores (p < 0.01).

The availability of the National Guidelines was similar at CHCs and hospitals (Table 1). Among the 155 CHCs participating in the knowledge survey, 74% had a copy of the National Guidelines. There was a similar mean score in the knowledge survey among staff having access to the National Guidelines at their CHC (28.7) and those not having such access (28.6), (p = 0.96). During 2005, 32% (131/412) of the knowledge survey respondents worked at a CHC where staff had not assisted in any deliveries, 49% (202/412) worked at a CHC where staff had assisted in 1 to 24 deliveries and 19% (79/412) worked at a CHC where the staff assisted in 25 to 92 deliveries. There was no association between the staff's level of knowledge and the number of deliveries at the corresponding CHC (p = 0.44).

Based on the results from the knowledge survey, the 12 districts were divided into two groups (the districts with the six highest and six lowest mean scores), resulting in two distinct geographical areas, designated here as the northeast districts (NED) and the southwest districts (SWD) (Fig. 1). NED consisted of 68 CHCs where staff had a mean score of 27.1 on the survey, while staff in the 87 CHCs in SWD achieved a mean score of 29.9 (p < 0.01). This distinct geographical division led us to analyse whether the use of health care services, neonatal death and other factors related to neonatal health also differed between the two areas. The two areas were different in all assessed health care outcomes. The NED had fewer pregnant women who attended three or more ANC visits, fewer families receiving a postnatal home visit, higher NMR and lower accessibility of National Guidelines than the CHCs in the SWD (Table 2). The two tertiary level hospitals in the province were both situated in the SWD (Fig. 1). Patients and health care personnel in the NED were, on average, three times farther from the tertiary hospitals than patients and personnel in the SWD (Table 2).

**Table 2:** Characteristics of the northeast districts (NED) and the southwest districts (SWD) of Quang Ninh province, Viet Nam, 2005

**Variable**	**NED**	**SWD**	**P-value**
Average distance to regional hospital (kilometres)	95	31	<0.01^1^

Average distance to provincial hospital (kilometres)	75	24	<0.01^1^

Neonatal mortality rate^a^	21.9	14.2	<0.01^2^

Percentage of pregnant women with at least three antenatal care visits	45.6	70.6	<0.01^2^

Percentage of live births receiving a postnatal home visit	48.5	56.7	<0.01^2^

Percentage of community health centres having the National Guidelines^b^	64.7	79.3	<0.05^2^

^a ^Number of deaths during the first 28 days of life per 1000 live births

^b ^*Guidelines of reproductive health *(2003) by the Ministry of Health in Viet Nam

^1 ^P-value derived from independent sample t-test comparing NED and SWD

^2^P-value derived from the χ^2 ^test comparing the NED and the SWD

## Discussion

Only 60% of the potential points in the knowledge survey were achieved, indicating that primary health care staff in the current province appears to have deficient knowledge regarding basic, evidence-based neonatal care. Geographical location was identified as a factor linked to staff knowledge on EBP. The level of knowledge was not associated with access to the National Guidelines or the volume of deliveries at community level, however.

By targeting the primary health care practitioners on duty at the time of data collection in the 12 districts, an acceptable coverage was reached (i.e. 63% of all staff individuals). The survey questions were based on recommendations in general guidelines [20,25] and further supported by the findings in a recently published systematic review of community-based interventions for perinatal health [26]. These sources emphasize the importance of the five neonatal topics targeted in our knowledge survey. The questionnaire was not complete in its coverage of issues within the five topics, but sufficient to provide an indication of the level of neonatal knowledge among primary health care staff. We therefore consider the present questionnaire to be a valid tool for measuring basic knowledge on EBP. Pilot tests of the survey questions further strengthened their validity. The basic character of the questions made it reasonable to expect that staff should be able to answer without help from books or guidelines. We chose to use multiple-choice questions because it was a feasible way to assess several areas of neonatal health. This knowledge assessment approach was also familiar to the respondents. Because we used several multiple-choice questions and a mix of single and multiple correct alternatives, the chance of guessing the correct answers was minimal [27]. However, there are limitations with this method that need to be considered when interpreting the result [28]. Most of the literature recommends constructing multiple-choice questions with only one correct answer [29]; still, the model we used is considered adequate when using a scoring system adapted to multiple answers [27]. We chose to give the same weight to all the survey questions (and topics). We also believe that the external validity is acceptable, since findings could reflect the situation in many other provinces in Viet Nam. The country has a uniform health care policy and structure, and even though Quang Ninh is a rather rich province it can be considered representative in terms of geography and demography [22]. Regarding the two districts not included in the survey, one district was similar in characteristics to the districts in the NED and the other similar in characteristics to the districts in the SWD. Including these districts in the survey would most likely not have changed the overall outcome. A weakness of the NeoKIP baseline study was the lack of socioeconomic mapping of the population, such as prevalence of poverty and ethnic minorities. We tried to collect data on socioeconomic factors from all the districts, but the information was incomplete and therefore not presented here. To get a deeper understanding of staff knowledge and the processes of knowledge translation prior to the planned intervention, focus group discussions were conducted with primary health care staff from some of the districts included in the survey. The findings will be published elsewhere.

Knowledge translation interventions are, too often, implemented without proper examination of the situation before and during the intervention [11]. Assessing the level of neonatal knowledge in this survey has helped us to understand if practitioners' awareness of current knowledge is an issue that needs to be addressed in the coming intervention. The staff performed well on some questions, but the overall findings must be judged as relatively poor, as the questions were limited to a basic level. The poor results on the questions about umbilical cord management indicate that CHCs lack a common strategy, despite concordant recommendations for cord care in the literature [20,25]. Furthermore, the respondents had higher scores on questions about initiating breastfeeding than duration of breastfeeding. This corresponds to how women in a previous study in Viet Nam responded to questions about breastfeeding [30] and might indicate a general lack of knowledge among health care workers on WHO's recommendations about duration of breastfeeding [31]. Another area assessed in the knowledge survey was immediate postnatal care. The results clearly demonstrate a lack of knowledge on the particular questions on this topic. This finding could be due to the suboptimal working conditions of the primary health care staff in comparison to the situation for hospital staff. For example, the availability of basic delivery equipment, such as mask and ambo, radiant heater, towels and Vitamin K_1_, were considerably lower at CHCs than in hospitals. Lack of resources can result in negative health care consequences. A recent study from Viet Nam reports vitamin K deficiency as a major problem in the region around Hanoi [32], a problem that is preventable by increased knowledge and use of Vitamin K prophylaxis.

There was no difference in knowledge between staff at CHCs with access to the National Guidelines and staff at CHCs lacking the guidelines. Despite availability of guidelines in three out of four CHCs, the recommendations do not seem to be fully known by the health care workers who participated in this study. This finding, which is consistent with previous research [33,34], suggests that access alone to the National Guidelines does not imply enhanced knowledge, indicating that passive dissemination of guidelines has limited impact. Additional methods reinforcing the implementation of these guidelines appear to be necessary [35,36]. There is a range of methods for active implementation of guidelines, including reminders, opinion leaders, interactive small group meetings, audit and feedback [35,37,38]. These methods are all suggested to be effective, but the circumstances under which they work best remain to be further evaluated. Intervention studies in the knowledge translation field are particularly required in developing countries [39]. The global need to advance this field of knowledge is great, as is the potential for clinical improvements in developing settings.

In addition to adequate knowledge and resources, the health care staff needs to have a certain level of clinical activity to be able to maintain competence and skills. For example, it is assumed that having too few deliveries in a health care institution results in difficulties in maintaining high standard delivery care [17]. Yet, in the current study no association was found between level of knowledge and the number of assisted deliveries at the community level. Most likely, the overall delivery activity was too low to identify any association.

Staff level of knowledge was associated with the geographical location of the CHCs, where the participating districts were clearly clustered into two distinct areas, with staff working in the north-east scoring lower than staff in the south-west part of the province. Unfortunately we lack detailed socioeconomic information at the district and community level, but the two geographical areas most likely differ in such demographics [18,22]. The NED, which is a more pronouncedly mountainous and rural area, can be assumed to have a larger proportion of poor people and inhabitants belonging to an ethnic minority group than the SWD. It is known that poorer segments of a population benefit less from public spending in health [40] and inequity is a complex and common problem within many developing countries [23]. The distribution of knowledge regarding neonatal practices in Quang Ninh province might be explained using Rogers's theory on the diffusion of innovations [41] and the inverse equity hypothesis postulated by Victora and colleagues [42]. New (health care) interventions first tend to be adopted by a subset of the population [41,43] and the inhabitants belonging to groups with higher socioeconomic status are initially reached more often, resulting in an increased gap between socioeconomic groups [42]. This gap, however, tends to narrow over time when the demands in the higher socioeconomic groups decrease.

Another potential causative factor to account for the association between knowledge and geographical location is the allocation of hospitals. The health care facilities at the primary and secondary level were evenly distributed, but the two tertiary hospitals were located in the south. In developing countries where infrastructure often is insufficient, factors such as long distance to hospitals and remote location are known to have implications for mortality [44]. People in the NED were, on average, three times farther from these hospitals than people in the SWD. Much of the health care expertise is located in the large hospitals, which are also centres for training and education. Rodgers et al. [45] emphasize opportunities for continuing education as a crucial factor for research utilization among health care staff, and a multi-country study by Victora and colleagues [46] showed lower uptake of a health intervention in poorer and more rural areas. Furthermore, Olade [16] describes several contextual barriers for research utilization among rural nurses, some of which are isolation and lack of knowledgeable professional colleagues. There are reasons to believe that a long distance between CHCs and tertiary level hospitals could be a barrier, both to knowledge translation and quality of care. Many contextual factors are considered to be linked to research utilization, but most studies have investigated only factors within organizations (e.g. working climate and access to research resources) [13,14]. In accordance with Andrews and Moon [15], we believe that geographical location of health care units is a contextual factor that deserves more attention. Looking upon staff level of knowledge through a geographical lens has helped us understand where certain efforts are primarily needed in the future.

The two geographical areas that had different levels of knowledge among health care staff were also found to differ in quality of care (ANC and postnatal home visits) and neonatal survival. Generally there was uncertainty among survey respondents as to why, when and by whom a home visit should be conducted, even though this is clearly described in the National Guidelines [20] and stated in other literature [7,47,48]. More than half of all families with a newborn child did not receive a home visit and, in accordance with the knowledge survey, staff members working in the NED were conducting fewer home visits than staff in the SWD. If home visits are neglected, there is an increased risk that severe infections that might arise a few days after birth are not detected. Such infections, in most cases, will need the attention of professional health care personnel [3,7], and the absence of home visits might therefore have severe consequences for the families. This is especially true for those families with a woman delivering at home, since a home visit of a midwife could be the family's only link to the health care system. Moreover, there was a 50% higher NMR in the NED than in the SWD, which strongly points to an inequity in neonatal survival, probably primarily because of differences in socioeconomic factors [23] and distance to health facilities. The difference in knowledge among health care staff might also contribute to the difference in neonatal mortality: the area with the lowest level of knowledge had the highest NMR. Evidently the difference in knowledge alone cannot explain the difference in NMR. Rather, this identified link between knowledge and neonatal mortality might provide one ingredient in a complex picture of potentially casual associations. Globally, the underuse of EBP is described as a major reason for high neonatal mortality [7]. Further, recent studies demonstrate that increased use of EBP resulted in improved neonatal care and reduced neonatal mortality [10,12]. Whether staff level of knowledge is a contributing cause to the inequities in quality of care and neonatal survival or an effect of differences in socioeconomic factors is open for further investigation and discussion. Tugwell and co-workers suggest an evidence-based framework for equity-oriented knowledge translation to incorporate issues on health equity [49]. This framework underlines the importance of identifying and prioritizing barriers as a base for choosing effective knowledge translation strategies for individuals belonging to different socioeconomic groups.

## Conclusion

Overall, the findings point to a rather low level of knowledge in neonatal care among the primary health care workers in a Vietnamese province. We also found that geographical location of a community health centre was associated with the level of knowledge. Two distinct geographical areas not only differed in staff knowledge but also proved to have major inequities in neonatal mortality and quality of neonatal care. These inequities were probably linked to socioeconomic differences. Although the findings indicate a complex web of associations involving knowledge, geography, demographic factors and neonatal outcomes, we believe staff knowledge and use of knowledge to be important and feasible factors to work on for improving the neonatal health situation in Viet Nam.

## Competing interests

The authors declare that they have no competing interests.

## Authors' contributions

LE and LW designed the study, with assistance of NTN, MM, LÅP and UE. NTN supervised data collection. LE cleaned data and performed the statistical analyses together with LW. LE was lead author in writing the manuscript, primarily assisted by LW. All authors have read and approved the final manuscript.

## Supplementary Material

Additional file 1 **Questionnaire on neonatal knowledge**. Questions used for the assessment of knowledge level on neonatal procedures among primary health care staff in Quang Ninh province, Viet Nam. Click here for file
