# Supplementary material for: Evidence-based practice in neonatal health: knowledge among primary health care staff in northern Viet Nam
Source: Hum Resour Health. 2009 Apr 24;7:36. doi: 10.1186/1478-4491-7-36 (PMC2678076; doi:10.1186/1478-4491-7-36)
Supplement: Additional file 1 — Questionnaire on neonatal knowledge. Questions used for the assessment of knowledge level on neonatal procedures among primary health care staff in Quang Ninh province, Viet Nam. [file 1478-4491-7-36-S1.doc]

| **Breastfeeding** |
| --- |
| 1. **When is it most appropriate to initiate breastfeeding after birth?**   a) Within the first hour, b) 1-6 h after birth, c) 6-12 h, d) More than 12 h after birth, e) I have no opinion, f) Other. |
| 1. **If the mother experiences that she has not enough breast milk during the first few days after delivery, what advice do you give her?**   a) To give formula while waiting for milk production to start, b) To breastfeed more frequently, c) To give rice water, herbal fluids or honey water while waiting for milk production to start, d) Advise the mother to ask someone else to breastfeed her baby: for example, a neighbour who has enough breast milk, e) I have no opinion, f) Other. |
| 1. **For how long should a mother continue to exclusively breastfeed her child?**   a) 1 month, b) 2 months, c) 4 months, d) 6 months, e) More than 6 months, f) I have no opinion, g) Other. |
| 1. **According to you, after how long should a mother stop breastfeeding her child completely?**   a) 6 months, b) 12 months, c) 18 months, d) 24 months, e) More than 2 years, f) I have no opinion, g) Other |
|  |
| **Immediate postnatal care** |
| 1. **Shortly after birth a healthy newborn baby should be able to: cry loud, have a pink colour to the skin, breathe evenly and have a respiratory rate of 40 – 60 breaths per minute. How would you take care of a baby that does not have these signs shortly after birth?**   a) Dry baby with cloth, b) Use bag and face mask to help baby with respiration, c) Suction of nose and mouth if necessary, d) Slapping baby, e) Pour cold water over baby, f) I have no opinion, g) Other. |
| 1. **What can be done to prevent newborn children from bleeding?**   a) Breastfeeding the child, b) Not necessary to give any drugs, c) Give Vitamin K, d) Give Vitamin K_1_, e) I have no opinion, f) Other. |
| 1. **Do you know what dose of Vitamin K_1_ to give to a newborn baby according to recommendations in the National Guidelines?**   a) 0.5 mg, b) 1 mg, c) 2 mg, d) 5 mg, e) 10 mg, f) I have no opinion, g) Other. |
|  |
| **Infection management** |
| 1. **A newborn child can get eye infections after delivery. Which of the following alternatives would you use to prevent this from occurring?**   a) Do not apply anything, b) Apply breast milk in the babies’ eyes, c) Clean eyes with sterile water, d) Apply eye drops (silver nitrate) after cleaning eyes, e) I have no opinion, f) Other. |
| 1. **Taking care of the umbilical cord of a newborn after delivery is important. Which of the following alternatives would you consider as important?**   a) Always clean your hands before touching the cord, b) Cut the cord with a clean instrument (for example, a razor blade), c) Use any sharp instrument for cutting the cord, d) After cutting the cord, apply traditional herbs/medicines, e) Always put a bandage around the cord, f) I have no opinion, g) Other. |
| 1. **What approach would you use to handle an umbilical cord that has any of the following signs: bad smell, oozing blood, small rashes around the umbilical area?**   a) Leave to dry, b) Clean with water and soap, c) Clean with iodine solution, d)Apply antibiotic powder, e) Refer to hospital, f) I have no opinion, g) Other. |
|  |
| **Temperature/Low birth weight** |
| 1. **What is, in your opinion, the best way to stabilise the temperature of a newborn baby?**   a) Bathing the baby in water of appropriate temperature, b) By putting on clothes and cover head, c) Having the baby skin-to-skin with her/his mother, d) Keep the baby in a room with a temperature of 28-30°C, e) Have the baby close to heat (radiator, fire, etc.), f) I have no opinion, g) Other. |
| 1. **What is the definition of low birth weight for newborn child?**   a) Weight is less than 3000 grams, b) Weight is less than 2500 grams, c) Weight is less than 1500 grams, d) Weight is less than 1000 grams, e) I have no opinion, f) Other |
| 1. **What alternatives are important when taking care of a low birth weight baby?**   a) Bath the baby often, b) Start breastfeeding early and frequently, c) Keep the child warm, d) Prevent infection from developing, e) I have no opinion, f) Other. |
|  |
| **Home visits** |
| 1. **Why is it important to conduct home visits to mothers and their newborn babies after delivery?**   a) To ask and examine the mother about her physical and psychological status, b) To ask the mother about the baby’s behaviour  (feeding, stools and urination, umbilical cord condition, etc.), c) Examine the baby regarding icterus, weight, skin, umbilical cord,  temperature, etc., d) I have no opinion, e) Other |
| 1. **When is the optimal time for health care personal to conduct the first home visit to a mother and her baby after delivery?**   a) Not important with home visits, b) During the first 3 days, c) Between day 3 and day 7, d) Between day 7 and day 14, e) I have no opinion, f) Other. |
| 1. **Who should conduct a home visit to a mother and her newborn baby after delivery?**   a) No one, b) A village health worker, c) A nurse, d) A midwife, e) A doctor, f) I have no opinion, g) Other |
